# [2-((*R*)-{2-[(*S*)-1-Benzylpyrrolidin-2-ylcarbonylazanidyl]­phen­yl}(phen­yl)methyl­idene­amino)-4-hy­droxy­butano­ato-κ^4^ *N*,*N*′,*N*′′,*O* ^1^]nickel(II) toluene disolvate

**DOI:** 10.1107/S1600536811031059

**Published:** 2011-08-17

**Authors:** Zdeňka Padělková, Alexander Popkov, Milan Nádvorník

**Affiliations:** a Department of General and Inorganic Chemistry, Faculty of Chemical Technology, University of Pardubice, Studentská 573, 53210 Pardubice, Czech Republic; b Department of Chemistry and Environmental Technology, Faculty of Technology, Tomas Bata University in Zlín, nám T. G. Masaryka 275, 762 72 Zlín, Czech Republic

## Abstract

The central Ni atom in the title compound, [Ni(C_29_H_29_N_3_O_4_)]·2C_7_H_8_, is coordinated in a distorted square-planar environment by three N atoms [Ni—N = 1.942 (3), 1.843 (3) and 1.853 (3) Å] and one O atom [1.868 (3) Å] of the tetradentate ligand. The conformation of the hy­droxy­butano­ate side chain is controlled by an inter­molecular hydrogen bond.

## Related literature

For the synthesis of similar complexes and their potential use as radiotracers, see: Bourdier *et al.* (2011 ▶); Fasth & Långström (1990 ▶); Kožíšek *et al.* (2004 ▶); Langer *et al.* (2007 ▶); Popkov & Breza (2010 ▶); Popkov *et al.* (2005 ▶, 2008 ▶, 2010 ▶); Nádvorník *et al.* (2008 ▶).
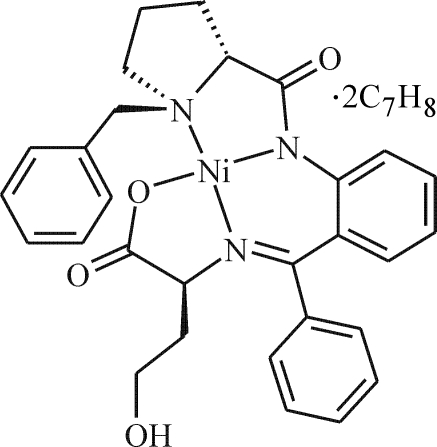


## Experimental

### 

#### Crystal data

[Ni(C_29_H_29_N_3_O_4_)]·2C_7_H_8_
*M* *_r_* = 726.53
Orthorhombic, 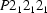

*a* = 11.2660 (14) Å
*b* = 12.8570 (9) Å
*c* = 24.527 (3) Å
*V* = 3552.7 (6) Å^3^
*Z* = 4
Mo *K*α radiation
μ = 0.60 mm^−1^
*T* = 150 K
0.36 × 0.23 × 0.20 mm

#### Data collection

Bruker–Nonius KappaCCD area-detector diffractometer
Absorption correction: gaussian (Coppens, 1970 ▶) *T*_min_ = 0.852, *T*_max_ = 0.924
21133 measured reflections
7165 independent reflections
6170 reflections with *I* > 2σ(*I*)
*R*_int_ = 0.103

#### Refinement

*R*[*F* ^2^ > 2σ(*F* ^2^)] = 0.056
*wR*(*F* ^2^) = 0.142
*S* = 1.01
7165 reflections
461 parameters
H-atom parameters constrained
Δρ_max_ = 0.41 e Å^−3^
Δρ_min_ = −0.44 e Å^−3^
Absolute structure: Flack (1983 ▶), 3089 Friedel pairs
Flack parameter: 0.000 (17)

### 

Data collection: *COLLECT* (Hooft, 1998 ▶) and *DENZO* (Otwinowski & Minor, 1997 ▶); cell refinement: *COLLECT* and *DENZO*; data reduction: *COLLECT* and *DENZO*; program(s) used to solve structure: *SIR92* (Altomare *et al.*, 1994 ▶); program(s) used to refine structure: *SHELXL97* (Sheldrick, 2008 ▶); molecular graphics: *PLATON* (Spek, 2009 ▶); software used to prepare material for publication: *SHELXL97*.

## Supplementary Material

Crystal structure: contains datablock(s) I, global. DOI: 10.1107/S1600536811031059/im2308sup1.cif

Structure factors: contains datablock(s) I. DOI: 10.1107/S1600536811031059/im2308Isup2.hkl

Additional supplementary materials:  crystallographic information; 3D view; checkCIF report

## Figures and Tables

**Table 1:** Hydrogen-bond geometry (Å, °)

*D*—H⋯*A*	*D*—H	H⋯*A*	*D*⋯*A*	*D*—H⋯*A*
O2—H2⋯O3^i^	0.82	1.94	2.720 (5)	159

Symmetry code: (i) 
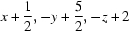
.

**Fig. 1.**
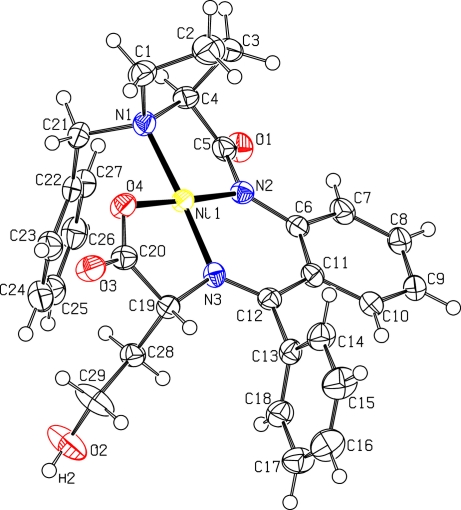
View of the title compound with displacement ellipsoids shown at the 50% probability level. H atoms are shown with arbitrary radii.

**Fig. 2.**
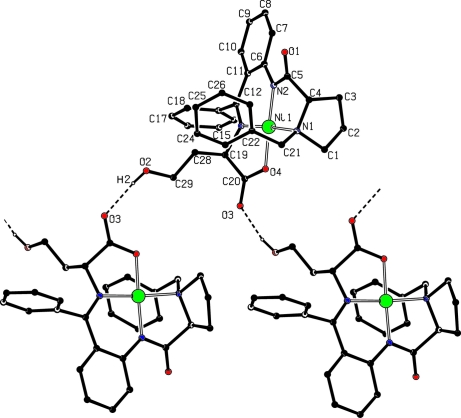
View of the motif of the crystal structure with hydrogen bonds indicated as dashed lines.

## supplementary crystallographic information

### Comment

Substituted Ni(II) complexes of Schiff bases derived from *(S)*-*N*-(2-benzoylphenyl)-1-benzylpyrrolidine-2-carboxamide and α-amino acids are intermediates for the synthesis of radiotracers for positron emission tomography (Fasth & Långström 1990, Popkov *et al.*, 2010). In the search for efficient and cheap radiotracers new approaches employing ω-labelled amino acids are being developed (Bourdier *et al.*, 2011). We published the first structure of a complex bearing a hydroxy group in ω-position of the amino acid fragment side chain (Popkov *et al.*, 2008), which was used for MP2 modelling and topological QTAIM analysis of reactivity of the complexes in alkylation reactions (Popkov & Breza, 2010). The absolute configuration of the chiral centres of this diastereomer is *SS*. Due to our interest in chiral nickel (II) complexes suitable for charge density studies (Kožíšek *et al.*, 2004), we intended to compare charge densities of two diastereomers of the same complex. In this communication we describe structure of the *SR*-diastereomer {2-[*(R)*-({2-*(S)*-1- benzylpyrrolidine-2-carboxamido]phenyl}(phenyl)methyleneamino]-4- hydroxybutanoato-κ*N-4,N',N'',O*} nickel(II) (Ni(II) complex of the Schiff base from *(S)*-*N*-(2-benzoylphenyl)-1-benzylpyrrolidine-2-carboxamide and *(R)*-2-amino-4-hydroxybutanoic acid), which is a candidate for charge density measurement. Structures of the two diastereomers differ a lot. Like in complexes derived from quaternary α-amino acids (Langer *et al.*, 2007) in the *SR*-diastereomer steric repulsion of the benzyl group and the side chain is very strong. It compensates steric factors which distort the coordination plane of the *SS*-complex and the *SR*-complex is approaching an ideal square-planar coordination. Unlike the *SS* -diastereomer where the hydrogen bond O4—H4AW···O3 is intramolecular, in the *SR*-complex a bond O2—H2···O3 is intermolecular. In both diastereomers the benzyl group is in apical position towards the nickel atom.

### Experimental

{2-[*(R)*-({2-*(S)*-1-benzylpyrrolidine-2-carboxamido]phenyl}(phenyl)methyleneamino]-4-hydroxybutanoato-κ*N-4,N',N'',O*} nickel(II) was chromatographically isolated as the minor diastereomer (4% yield) in the synthesis of {2-[*(S)*-({2-*(S)*-1-benzylpyrrolidine-2-carboxamido]phenyl} (phenyl)methylene)amino]-4-hydroxybutanoato-κ*N-4,N',N'',O*} nickel(II) (Popkov *et al.*, 2008). Crystals suitable for X-ray diffraction were prepared by crystallization from toluene-methanol (2:1) by slow evaporation at room temperature.

### Refinement

All hydrogen atoms were discernible in the difference electron density map. However, all hydrogen atoms were situated into idealized positions and refined riding on their parent C or O atoms, with O—H = 0.82 Å, C—H = 0.97 Å for methylene, 0.96 Å for methine, 0.93 Å for aromatic H atoms, with U(H) = 1.2U*_eq_*(O) for the alcohol and U(H) = 1.5U*_eq_*(C) for other H atoms, respectively.

### Crystal data

**Table:**

[Ni(C_29_H_29_N_3_O_4_)]·2C_7_H_8_	*F*(000) = 1536
*M**_r_* = 726.53	*D*_x_ = 1.358 Mg m^−^^3^
Orthorhombic, *P*2_1_2_1_2_1_	Mo *K*α radiation, λ = 0.71073 Å
Hall symbol: P 2ac 2ab	Cell parameters from 21280 reflections
*a* = 11.2660 (14) Å	θ = 1–26.5°
*b* = 12.8570 (9) Å	µ = 0.60 mm^−^^1^
*c* = 24.527 (3) Å	*T* = 150 K
*V* = 3552.7 (6) Å^3^	Block, red
*Z* = 4	0.36 × 0.23 × 0.20 mm

### Data collection

**Table:**

Bruker–Nonius KappaCCD area-detector diffractometer	7165 independent reflections
Radiation source: fine-focus sealed tube	6170 reflections with *I* > 2σ(*I*)
graphite	*R*_int_ = 0.103
Detector resolution: 9.091 pixels mm^-1^	θ_max_ = 26.5°, θ_min_ = 2.0°
φ and ω scans to fill the Ewald sphere	*h* = −13→14
Absorption correction: gaussian (Coppens, 1970)	*k* = −15→16
*T*_min_ = 0.852, *T*_max_ = 0.924	*l* = −27→30
21133 measured reflections	

### Refinement

**Table:**

Refinement on *F*^2^	Secondary atom site location: difference Fourier map
Least-squares matrix: full	Hydrogen site location: inferred from neighbouring sites
*R*[*F*^2^ > 2σ(*F*^2^)] = 0.056	H-atom parameters constrained
*wR*(*F*^2^) = 0.142	*w* = 1/[σ^2^(*F*_o_^2^) + (0.0456*P*)^2^ + 5.8181*P*] where *P* = (*F*_o_^2^ + 2*F*_c_^2^)/3
*S* = 1.01	(Δ/σ)_max_ = 0.001
7165 reflections	Δρ_max_ = 0.41 e Å^−^^3^
461 parameters	Δρ_min_ = −0.44 e Å^−^^3^
0 restraints	Absolute structure: Flack (1983), 3089 Friedel pairs
Primary atom site location: structure-invariant direct methods	Flack parameter: 0.000 (17)

### Special details

**Table:**

Geometry. All esds (except the esd in the dihedral angle between two l.s. planes) are estimated using the full covariance matrix. The cell esds are taken into account individually in the estimation of esds in distances, angles and torsion angles; correlations between esds in cell parameters are only used when they are defined by crystal symmetry. An approximate (isotropic) treatment of cell esds is used for estimating esds involving l.s. planes.
Refinement. Refinement of F^2^ against ALL reflections. The weighted R-factor wR and goodness of fit S are based on F^2^, conventional R-factors R are based on F, with F set to zero for negative F^2^. The threshold expression of F^2^ > 2sigma(F^2^) is used only for calculating R-factors(gt) etc. and is not relevant to the choice of reflections for refinement. R-factors based on F^2^ are statistically about twice as large as those based on F, and R- factors based on ALL data will be even larger.

### Fractional atomic coordinates and isotropic or equivalent isotropic displacement parameters (Å^2^)

**Table:**

	*x*	*y*	*z*	*U*_iso_*/*U*_eq_	
Ni1	0.41796 (4)	0.96012 (4)	0.967796 (19)	0.02409 (13)	
O4	0.4291 (3)	1.1043 (2)	0.97576 (11)	0.0313 (6)	
N3	0.5515 (3)	0.9687 (3)	0.92463 (12)	0.0243 (6)	
N1	0.2795 (3)	0.9604 (3)	1.01498 (12)	0.0276 (7)	
N2	0.4009 (3)	0.8177 (2)	0.95941 (13)	0.0254 (7)	
C12	0.5905 (4)	0.9023 (3)	0.88878 (14)	0.0252 (8)	
C22	0.4189 (4)	0.9437 (3)	1.09551 (14)	0.0308 (9)	
C5	0.3250 (4)	0.7761 (3)	0.99831 (16)	0.0292 (9)	
C28	0.7135 (3)	1.0520 (3)	0.97481 (17)	0.0287 (8)	
H28A	0.7561	0.9891	0.9652	0.034*	
H28B	0.6782	1.0410	1.0104	0.034*	
O3	0.5443 (3)	1.2393 (2)	0.95379 (13)	0.0366 (7)	
O1	0.3252 (3)	0.6883 (2)	1.01642 (12)	0.0355 (7)	
C20	0.5239 (4)	1.1452 (3)	0.95497 (16)	0.0297 (9)	
C4	0.2299 (4)	0.8524 (3)	1.01382 (16)	0.0285 (9)	
H4	0.1964	0.8344	1.0495	0.034*	
C9	0.5605 (4)	0.6291 (4)	0.83845 (17)	0.0336 (10)	
H9	0.5933	0.5884	0.8110	0.040*	
C19	0.6139 (3)	1.0688 (3)	0.93337 (16)	0.0250 (8)	
H19	0.6468	1.0939	0.8988	0.030*	
C23	0.5277 (4)	0.9944 (4)	1.09517 (18)	0.0384 (11)	
H23	0.5328	1.0594	1.0787	0.046*	
C21	0.3124 (4)	0.9958 (3)	1.07169 (16)	0.0330 (10)	
H21A	0.2453	0.9836	1.0956	0.040*	
H21B	0.3268	1.0702	1.0709	0.040*	
C11	0.5450 (4)	0.7964 (3)	0.88514 (15)	0.0255 (8)	
C6	0.4583 (3)	0.7542 (3)	0.92145 (15)	0.0250 (8)	
C8	0.4775 (4)	0.5876 (3)	0.87468 (18)	0.0349 (10)	
H8	0.4549	0.5183	0.8715	0.042*	
C13	0.6839 (4)	0.9361 (3)	0.84898 (16)	0.0278 (9)	
O2	0.8883 (3)	1.1086 (3)	1.01775 (18)	0.0631 (12)	
H2	0.9414	1.1522	1.0182	0.076*	
C7	0.4286 (4)	0.6479 (3)	0.91515 (17)	0.0323 (9)	
H7	0.3744	0.6182	0.9391	0.039*	
C18	0.8040 (4)	0.9271 (4)	0.85954 (17)	0.0359 (10)	
H18	0.8300	0.8955	0.8915	0.043*	
C3	0.1311 (4)	0.8533 (4)	0.97001 (19)	0.0373 (10)	
H3A	0.0555	0.8329	0.9856	0.045*	
H3B	0.1502	0.8061	0.9404	0.045*	
C27	0.4135 (5)	0.8469 (4)	1.12086 (16)	0.0364 (10)	
H27	0.3416	0.8114	1.1218	0.044*	
C10	0.5920 (4)	0.7317 (3)	0.84435 (15)	0.0304 (8)	
H10	0.6476	0.7593	0.8204	0.037*	
C2	0.1265 (5)	0.9646 (5)	0.94919 (19)	0.0473 (12)	
H2A	0.0451	0.9855	0.9423	0.057*	
H2B	0.1718	0.9723	0.9158	0.057*	
C14	0.6456 (4)	0.9796 (4)	0.79975 (16)	0.0384 (11)	
H14	0.5648	0.9843	0.7923	0.046*	
C25	0.6204 (4)	0.8544 (4)	1.14307 (18)	0.0402 (11)	
H25	0.6874	0.8240	1.1584	0.048*	
C1	0.1801 (4)	1.0286 (4)	0.99426 (18)	0.0382 (10)	
H1A	0.2103	1.0943	0.9806	0.046*	
H1B	0.1225	1.0421	1.0228	0.046*	
C15	0.7278 (5)	1.0161 (4)	0.7621 (2)	0.0490 (13)	
H15	0.7022	1.0449	0.7294	0.059*	
C26	0.5126 (5)	0.8026 (4)	1.14463 (19)	0.0405 (11)	
H26	0.5067	0.7381	1.1616	0.049*	
C17	0.8863 (4)	0.9650 (4)	0.82149 (18)	0.0422 (11)	
H17	0.9672	0.9609	0.8288	0.051*	
C32	0.8207 (5)	0.5005 (5)	0.7516 (2)	0.0555 (14)	
H32	0.8700	0.5559	0.7608	0.067*	
C31	0.7956 (7)	0.4249 (5)	0.7887 (2)	0.0687 (18)	
H31	0.8328	0.4267	0.8226	0.082*	
C16	0.8479 (5)	1.0092 (4)	0.7733 (2)	0.0502 (14)	
H16	0.9027	1.0348	0.7483	0.060*	
C24	0.6281 (5)	0.9516 (5)	1.11859 (19)	0.0475 (12)	
H24	0.6996	0.9876	1.1179	0.057*	
C29	0.7998 (5)	1.1393 (4)	0.9789 (2)	0.0536 (15)	
H29A	0.7602	1.2023	0.9908	0.064*	
H29B	0.8361	1.1523	0.9437	0.064*	
C30	0.7161 (6)	0.3442 (5)	0.7772 (2)	0.0616 (16)	
C35	0.6702 (6)	0.3399 (5)	0.7253 (3)	0.0666 (17)	
H35	0.6196	0.2857	0.7158	0.080*	
C33	0.7705 (6)	0.4941 (5)	0.6997 (2)	0.0623 (16)	
H33	0.7887	0.5437	0.6734	0.075*	
C34	0.6963 (6)	0.4156 (6)	0.6886 (3)	0.0674 (18)	
H34	0.6629	0.4119	0.6539	0.081*	
C42	0.7867 (9)	0.7455 (5)	0.6131 (3)	0.078 (2)	
H42	0.7321	0.7470	0.5847	0.094*	
C38	0.8331 (8)	0.7707 (5)	0.7070 (3)	0.076 (2)	
H38	0.8089	0.7879	0.7420	0.092*	
C39	0.9515 (8)	0.7540 (6)	0.6966 (3)	0.081 (2)	
H39	1.0061	0.7560	0.7250	0.097*	
C41	0.9073 (9)	0.7280 (5)	0.6024 (3)	0.082 (2)	
H41	0.9318	0.7110	0.5674	0.098*	
C37	0.7498 (8)	0.7643 (5)	0.6665 (3)	0.074 (2)	
C40	0.9906 (8)	0.7345 (6)	0.6443 (3)	0.084 (2)	
H40	1.0710	0.7251	0.6373	0.101*	
C36	0.6855 (11)	0.2664 (8)	0.8205 (3)	0.120 (4)	
H36A	0.7492	0.2173	0.8240	0.144*	
H36B	0.6744	0.3020	0.8545	0.144*	
H36C	0.6138	0.2303	0.8110	0.144*	
C43	0.6229 (9)	0.7779 (9)	0.6775 (4)	0.113 (3)	
H43A	0.6125	0.8359	0.7017	0.136*	
H43B	0.5812	0.7909	0.6441	0.136*	
H43C	0.5920	0.7162	0.6943	0.136*	

### Atomic displacement parameters (Å^2^)

**Table:**

	*U*^11^	*U*^22^	*U*^33^	*U*^12^	*U*^13^	*U*^23^
Ni1	0.0244 (2)	0.0219 (2)	0.0260 (2)	−0.0005 (2)	0.0026 (2)	−0.0001 (2)
O4	0.0314 (15)	0.0260 (13)	0.0366 (15)	−0.0005 (13)	0.0081 (14)	−0.0015 (12)
N3	0.0293 (17)	0.0172 (14)	0.0264 (14)	0.0012 (14)	−0.0020 (12)	−0.0002 (13)
N1	0.0286 (16)	0.0262 (16)	0.0278 (15)	0.0026 (16)	−0.0002 (12)	−0.0028 (15)
N2	0.0241 (17)	0.0251 (16)	0.0269 (16)	−0.0012 (14)	0.0002 (14)	0.0004 (13)
C12	0.0227 (18)	0.0280 (19)	0.0249 (17)	0.0030 (17)	−0.0036 (16)	0.0019 (15)
C22	0.033 (2)	0.036 (2)	0.0242 (17)	0.000 (2)	0.0026 (17)	−0.0118 (16)
C5	0.029 (2)	0.034 (2)	0.0252 (19)	−0.0097 (19)	0.0021 (16)	−0.0007 (17)
C28	0.0259 (18)	0.0228 (19)	0.037 (2)	−0.0020 (16)	0.0009 (16)	−0.0007 (18)
O3	0.0333 (15)	0.0251 (14)	0.0513 (18)	−0.0014 (13)	0.0081 (13)	0.0002 (13)
O1	0.0424 (17)	0.0281 (15)	0.0361 (16)	−0.0066 (14)	0.0027 (13)	0.0037 (12)
C20	0.028 (2)	0.028 (2)	0.033 (2)	0.0009 (17)	0.0005 (16)	0.0019 (17)
C4	0.0244 (19)	0.032 (2)	0.0289 (19)	−0.0059 (18)	0.0058 (16)	0.0022 (17)
C9	0.030 (2)	0.036 (2)	0.034 (2)	0.0021 (19)	−0.0030 (17)	−0.0102 (18)
C19	0.027 (2)	0.0195 (18)	0.0288 (18)	−0.0062 (15)	0.0059 (15)	0.0010 (14)
C23	0.042 (3)	0.043 (3)	0.031 (2)	−0.006 (2)	0.0013 (19)	−0.0049 (19)
C21	0.034 (2)	0.034 (2)	0.030 (2)	−0.0008 (19)	0.0052 (18)	−0.0081 (17)
C11	0.0265 (19)	0.0271 (19)	0.0227 (17)	−0.0013 (16)	−0.0039 (15)	0.0017 (15)
C6	0.0222 (17)	0.0238 (19)	0.0289 (19)	−0.0008 (16)	−0.0035 (15)	−0.0009 (16)
C8	0.034 (2)	0.030 (2)	0.041 (2)	−0.0021 (19)	−0.0030 (19)	−0.0095 (19)
C13	0.032 (2)	0.0209 (19)	0.0301 (19)	−0.0032 (17)	0.0037 (17)	−0.0023 (15)
O2	0.051 (2)	0.0374 (18)	0.101 (3)	−0.0065 (17)	−0.039 (2)	−0.0001 (19)
C7	0.033 (2)	0.028 (2)	0.036 (2)	−0.0074 (19)	−0.0013 (19)	−0.0014 (16)
C18	0.034 (2)	0.045 (3)	0.029 (2)	−0.001 (2)	0.0042 (18)	0.0009 (18)
C3	0.0254 (19)	0.051 (3)	0.035 (2)	−0.0065 (19)	0.000 (2)	−0.003 (2)
C27	0.037 (2)	0.040 (2)	0.032 (2)	−0.001 (2)	0.000 (2)	−0.0053 (18)
C10	0.028 (2)	0.034 (2)	0.0292 (19)	−0.001 (2)	−0.0037 (17)	−0.0026 (16)
C2	0.042 (3)	0.059 (3)	0.041 (2)	0.007 (3)	−0.006 (2)	0.004 (3)
C14	0.038 (2)	0.050 (3)	0.027 (2)	0.003 (2)	0.0023 (18)	0.003 (2)
C25	0.042 (3)	0.047 (3)	0.031 (2)	0.006 (2)	−0.0071 (19)	−0.008 (2)
C1	0.032 (2)	0.037 (2)	0.045 (2)	0.005 (2)	0.0045 (19)	−0.004 (2)
C15	0.051 (3)	0.060 (3)	0.036 (2)	0.001 (3)	0.007 (2)	0.015 (2)
C26	0.051 (3)	0.039 (3)	0.031 (2)	0.004 (2)	−0.001 (2)	−0.001 (2)
C17	0.033 (2)	0.049 (3)	0.046 (2)	−0.004 (2)	0.0067 (18)	0.003 (2)
C32	0.048 (3)	0.062 (4)	0.057 (3)	−0.008 (3)	0.002 (3)	−0.013 (3)
C31	0.093 (5)	0.068 (4)	0.045 (3)	0.007 (4)	−0.010 (3)	−0.013 (3)
C16	0.053 (3)	0.058 (3)	0.040 (3)	−0.009 (3)	0.014 (2)	0.010 (2)
C24	0.046 (3)	0.058 (3)	0.039 (2)	−0.011 (3)	0.001 (2)	−0.015 (2)
C29	0.044 (3)	0.041 (3)	0.076 (4)	−0.014 (2)	−0.031 (3)	0.014 (3)
C30	0.075 (4)	0.052 (3)	0.059 (3)	−0.004 (3)	0.007 (3)	−0.002 (3)
C35	0.065 (4)	0.065 (4)	0.070 (4)	−0.019 (3)	−0.010 (3)	−0.001 (3)
C33	0.066 (4)	0.066 (4)	0.054 (3)	−0.013 (3)	0.004 (3)	−0.001 (3)
C34	0.057 (4)	0.091 (5)	0.054 (3)	−0.016 (3)	−0.014 (3)	0.004 (3)
C42	0.119 (7)	0.060 (4)	0.056 (4)	0.000 (4)	−0.008 (4)	−0.002 (3)
C38	0.113 (6)	0.057 (4)	0.059 (4)	−0.008 (4)	−0.002 (4)	−0.003 (3)
C39	0.107 (6)	0.081 (5)	0.056 (4)	−0.009 (5)	−0.010 (4)	−0.002 (4)
C41	0.135 (7)	0.054 (4)	0.057 (4)	−0.020 (5)	0.003 (5)	−0.006 (3)
C37	0.110 (6)	0.053 (4)	0.059 (4)	0.001 (4)	−0.011 (4)	−0.003 (3)
C40	0.116 (7)	0.072 (5)	0.064 (4)	−0.018 (5)	0.005 (4)	−0.003 (4)
C36	0.179 (11)	0.097 (7)	0.084 (6)	−0.008 (7)	0.013 (6)	0.030 (5)
C43	0.126 (9)	0.130 (9)	0.083 (5)	0.025 (7)	−0.017 (5)	−0.018 (5)

### Geometric parameters (Å, °)

**Table:**

Ni1—N3	1.843 (3)	C27—H27	0.9299
Ni1—N2	1.853 (3)	C10—H10	0.9299
Ni1—O4	1.868 (3)	C2—C1	1.504 (7)
Ni1—N1	1.942 (3)	C2—H2A	0.9699
O4—C20	1.294 (5)	C2—H2B	0.9700
N3—C12	1.302 (5)	C14—C15	1.390 (7)
N3—C19	1.482 (5)	C14—H14	0.9300
N1—C4	1.498 (5)	C25—C26	1.385 (7)
N1—C1	1.510 (5)	C25—C24	1.388 (8)
N1—C21	1.510 (5)	C25—H25	0.9301
N2—C5	1.388 (5)	C1—H1A	0.9700
N2—C6	1.396 (5)	C1—H1B	0.9700
C12—C11	1.458 (6)	C15—C16	1.383 (8)
C12—C13	1.500 (5)	C15—H15	0.9300
C22—C23	1.389 (6)	C26—H26	0.9301
C22—C27	1.392 (6)	C17—C16	1.381 (7)
C22—C21	1.493 (6)	C17—H17	0.9300
C5—O1	1.213 (5)	C32—C31	1.361 (9)
C5—C4	1.502 (6)	C32—C33	1.395 (8)
C28—C29	1.487 (6)	C32—H32	0.9300
C28—C19	1.530 (5)	C31—C30	1.400 (9)
C28—H28A	0.9699	C31—H31	0.9301
C28—H28B	0.9699	C16—H16	0.9301
O3—C20	1.232 (5)	C24—H24	0.9300
C20—C19	1.508 (6)	C29—H29A	0.9701
C4—C3	1.547 (6)	C29—H29B	0.9700
C4—H4	0.9798	C30—C35	1.374 (9)
C9—C10	1.374 (6)	C30—C36	1.500 (9)
C9—C8	1.395 (6)	C35—C34	1.359 (9)
C9—H9	0.9300	C35—H35	0.9300
C19—H19	0.9800	C33—C34	1.338 (9)
C23—C24	1.383 (7)	C33—H33	0.9301
C23—H23	0.9300	C34—H34	0.9299
C21—H21A	0.9699	C42—C41	1.402 (12)
C21—H21B	0.9700	C42—C37	1.393 (10)
C11—C10	1.405 (6)	C42—H42	0.9301
C11—C6	1.429 (5)	C38—C37	1.369 (10)
C6—C7	1.415 (5)	C38—C39	1.375 (11)
C8—C7	1.375 (6)	C38—H38	0.9300
C8—H8	0.9299	C39—C40	1.378 (10)
C13—C18	1.382 (6)	C39—H39	0.9300
C13—C14	1.399 (6)	C41—C40	1.394 (11)
O2—C29	1.434 (6)	C41—H41	0.9300
O2—H2	0.8199	C37—C43	1.466 (12)
C7—H7	0.9299	C40—H40	0.9300
C18—C17	1.403 (6)	C36—H36A	0.9601
C18—H18	0.9300	C36—H36B	0.9601
C3—C2	1.521 (7)	C36—H36C	0.9600
C3—H3A	0.9700	C43—H43A	0.9599
C3—H3B	0.9701	C43—H43B	0.9601
C27—C26	1.383 (7)	C43—H43C	0.9599
			
N3—Ni1—N2	94.59 (14)	C9—C10—H10	118.3
N3—Ni1—O4	86.88 (14)	C11—C10—H10	118.6
N2—Ni1—O4	177.89 (15)	C1—C2—C3	104.7 (4)
N3—Ni1—N1	176.16 (15)	C1—C2—H2A	110.8
N2—Ni1—N1	89.15 (14)	C3—C2—H2A	110.6
O4—Ni1—N1	89.41 (14)	C1—C2—H2B	110.7
C20—O4—Ni1	114.7 (3)	C3—C2—H2B	111.2
C12—N3—C19	120.5 (3)	H2A—C2—H2B	108.8
C12—N3—Ni1	128.6 (3)	C15—C14—C13	120.2 (5)
C19—N3—Ni1	110.8 (2)	C15—C14—H14	120.0
C4—N1—C1	104.7 (3)	C13—C14—H14	119.9
C4—N1—C21	112.9 (3)	C26—C25—C24	120.0 (5)
C1—N1—C21	108.5 (3)	C26—C25—H25	119.8
C4—N1—Ni1	106.6 (2)	C24—C25—H25	120.2
C1—N1—Ni1	113.4 (2)	C2—C1—N1	103.2 (4)
C21—N1—Ni1	110.7 (3)	C2—C1—H1A	111.3
C5—N2—C6	121.2 (3)	N1—C1—H1A	111.1
C5—N2—Ni1	111.6 (3)	C2—C1—H1B	111.1
C6—N2—Ni1	127.1 (3)	N1—C1—H1B	110.9
N3—C12—C11	122.4 (4)	H1A—C1—H1B	109.1
N3—C12—C13	119.1 (3)	C16—C15—C14	119.9 (5)
C11—C12—C13	118.5 (3)	C16—C15—H15	120.1
C23—C22—C27	117.4 (4)	C14—C15—H15	120.0
C23—C22—C21	119.7 (4)	C27—C26—C25	119.9 (5)
C27—C22—C21	122.7 (4)	C27—C26—H26	119.9
O1—C5—N2	127.5 (4)	C25—C26—H26	120.3
O1—C5—C4	121.1 (4)	C16—C17—C18	120.3 (5)
N2—C5—C4	111.2 (3)	C16—C17—H17	119.7
C29—C28—C19	114.7 (3)	C18—C17—H17	120.0
C29—C28—H28A	108.8	C31—C32—C33	118.9 (6)
C19—C28—H28A	108.6	C31—C32—H32	120.6
C29—C28—H28B	108.5	C33—C32—H32	120.5
C19—C28—H28B	108.5	C32—C31—C30	121.8 (6)
H28A—C28—H28B	107.5	C32—C31—H31	119.1
O3—C20—O4	124.2 (4)	C30—C31—H31	119.1
O3—C20—C19	120.4 (4)	C15—C16—C17	120.2 (5)
O4—C20—C19	115.4 (3)	C15—C16—H16	119.7
N1—C4—C5	110.1 (3)	C17—C16—H16	120.0
N1—C4—C3	106.0 (3)	C23—C24—C25	119.2 (5)
C5—C4—C3	110.0 (3)	C23—C24—H24	120.2
N1—C4—H4	110.2	C25—C24—H24	120.7
C5—C4—H4	110.3	O2—C29—C28	107.0 (4)
C3—C4—H4	110.1	O2—C29—H29A	110.5
C10—C9—C8	118.2 (4)	C28—C29—H29A	110.5
C10—C9—H9	120.9	O2—C29—H29B	110.2
C8—C9—H9	120.8	C28—C29—H29B	110.2
N3—C19—C20	107.3 (3)	H29A—C29—H29B	108.5
N3—C19—C28	108.8 (3)	C35—C30—C31	117.3 (6)
C20—C19—C28	110.6 (3)	C35—C30—C36	122.8 (7)
N3—C19—H19	109.9	C31—C30—C36	119.8 (7)
C20—C19—H19	110.1	C34—C35—C30	120.2 (6)
C28—C19—H19	110.1	C34—C35—H35	120.2
C24—C23—C22	122.2 (5)	C30—C35—H35	119.6
C24—C23—H23	119.2	C34—C33—C32	119.0 (6)
C22—C23—H23	118.6	C34—C33—H33	120.8
C22—C21—N1	115.0 (3)	C32—C33—H33	120.2
C22—C21—H21A	108.4	C33—C34—C35	122.6 (6)
N1—C21—H21A	108.6	C33—C34—H34	118.6
C22—C21—H21B	108.4	C35—C34—H34	118.7
N1—C21—H21B	108.7	C41—C42—C37	119.5 (7)
H21A—C21—H21B	107.5	C41—C42—H42	120.2
C10—C11—C6	118.5 (4)	C37—C42—H42	120.2
C10—C11—C12	117.6 (4)	C37—C38—C39	121.5 (7)
C6—C11—C12	123.8 (4)	C37—C38—H38	119.0
N2—C6—C7	121.9 (4)	C39—C38—H38	119.6
N2—C6—C11	120.7 (3)	C38—C39—C40	120.6 (8)
C7—C6—C11	117.4 (4)	C38—C39—H39	119.9
C7—C8—C9	120.9 (4)	C40—C39—H39	119.5
C7—C8—H8	119.4	C42—C41—C40	120.3 (7)
C9—C8—H8	119.7	C42—C41—H41	120.0
C18—C13—C14	119.8 (4)	C40—C41—H41	119.7
C18—C13—C12	122.7 (4)	C38—C37—C42	119.1 (8)
C14—C13—C12	117.4 (4)	C38—C37—C43	121.8 (7)
C29—O2—H2	109.1	C42—C37—C43	119.0 (8)
C8—C7—C6	121.9 (4)	C39—C40—C41	118.8 (9)
C8—C7—H7	119.2	C39—C40—H40	120.4
C6—C7—H7	118.9	C41—C40—H40	120.8
C13—C18—C17	119.5 (4)	C30—C36—H36A	109.3
C13—C18—H18	120.2	C30—C36—H36B	109.2
C17—C18—H18	120.3	H36A—C36—H36B	109.5
C2—C3—C4	105.4 (4)	C30—C36—H36C	110.0
C2—C3—H3A	110.9	H36A—C36—H36C	109.5
C4—C3—H3A	110.9	H36B—C36—H36C	109.4
C2—C3—H3B	110.2	C37—C43—H43A	109.0
C4—C3—H3B	110.8	C37—C43—H43B	109.9
H3A—C3—H3B	108.7	H43A—C43—H43B	109.5
C26—C27—C22	121.4 (5)	C37—C43—H43C	109.5
C26—C27—H27	119.4	H43A—C43—H43C	109.5
C22—C27—H27	119.2	H43B—C43—H43C	109.5
C9—C10—C11	123.1 (4)		

### Hydrogen-bond geometry (Å, °)

**Table:**

*D*—H···*A*	*D*—H	H···*A*	*D*···*A*	*D*—H···*A*
O2—H2···O3^i^	0.82	1.94	2.720 (5)	159.

Symmetry codes: (i) *x*+1/2, −*y*+5/2, −*z*+2.
